# Hospital Meetings, &c.

**Published:** 1897-07-24

**Authors:** 


### HOSPITAL MEETINGS, &C.

### LAYING THE FOUNDATION STONE OF THE NEW CANCER WING OF THE MIDDLESEX HOSPITAL.

Her Royal Highness Princess Christian of Schleswig-Holstein laid the foundation stone of the new cancer wing of Middlesex Hospital. The Princess spread the cement with a silver trowel, which was afterwards presented to her in memory of the occasion, and helped to adjust the handsome stone, pronouncing in a clear voice the form prescribed.

The service, which had been written for the occasion, was conducted by the Bishop of Marlborough, aided by the choir of St. Andrew's, Well Street. It took place in a gaily coloured tent, which had been erected over the platform round the stone. The nurses of the hospital occupied the back of the platform, the Princess, her daughters, Princess Victoria and Princess Aribert of Anhalt on either hand, stood in the middle, and before them was the stone ready to be lowered into place. The tent was full to overflowing with invited guests. The Princess held a beautiful bouquet presented by Miss Thorold, the lady superintendent; the Princesses also had bouquets presented by nurses on the staff.

The Princess was attended by Colonel the Hon. C. G. Cornwallis Eliot, and was received by Sir Ralph Thompson, K.C.B., chairman of the weekly board, and the Hon. G. W. Spencer Lyttelton, C.B., Colonel Herbert Eaton, and the superintendent secretary, Mr. F. Clare Melhado. The following presentations were then made by the chairman: Mr. J. G. Noel, C.B., deputy-chairman of the weekly board; Mr. F. Hoare and Mr. G. J. Marjoribanks, treasurers; Dr. W. Cayley, senior physician; Mr. A. Clark, senior surgeon present; the Rev. W. G. Deighton, the chaplain; Mr. E. A. Fardon, the resident medical officer; Mr. F. Clare Melhado, the secretary superintendent; and Mr. Keith D. Young, the architect.

Amongst the guests were: Colonel the Hon. C. G. Cornwallis Eliot (in-waiting), Lord and Lady Amherst of Hackney, Viscount Gort, Sir Frank and Lady Lockwood, Sir Richard and Lady Douglas-Powell, Sir William Priestley, Sir John Hassard, Sir Elliott Lees, Bart., M.P., Colonel the Hon. C. G. Gathorne-Hardy and Lady Cicely, Sir Squire and Lady Bancroft, the Countess of Lathom, the Lady Ampthill, the Right Rev. the Bishop of Marlborough, the Rev. Canon Acheson, the Rev. W. F. Houldsworth, M.A., Dr. William Cayley (senior physician), Mr. Francis Hoare (treasurer), Mr. G. J. Marjoribanks (treasurer), Mr. E. A. Fardon, Mr. Keith Young (architect), Mr. James G. Noel (deputy chairman), Mr. Andrew Clark, F.R.C.S., Dr. Kingston Fowler, M.A., Mrs. Craufurd, Mr. F. Cavendish Bentinck, Mrs. Clare Melhado, Mr. G. Lawson, F.R.S., and Baron D'Erlanger.

Immediately after the ceremony the Princess and her suite left, whilst the guests were invited into the hospital for refreshments.

The plans for the new wing are not yet published, but there is to be accommodation for 36 patients, 12 nurses, and six domestics. The centre building is three storeys high; the two wings two. The basement is to be devoted to domestic offices; the first floor to the matron's rooms, nurses' dining-hall, linen cupboards, store-rooms, and servants' bed-rooms. The first storey contains two wards, a day-room, ward kitchen, baths, &c. The second, one ward, one isolation ward, nurses' dormitories, and bath room. The kitchen will occupy the top floor of the centre building, and communication will be facilitated by means of lifts. It is desired that the new wing shall be completed by next June. The cost is £15,000. £10,000 has been collected towards this, Mr. C. H. Berners granting the site on favourable terms.

### ASSOCIATION FOR THE ORAL INSTRUCTION OF THE DEAF AND DUMB.

A well-attended annual meeting of this association was held at the school, 11, Fitzroy Square, W., on Tuesday, 20th inst., Mr. L. Van Oven in the chair. The report, which was adopted on the motion of Dr. St. Clair Thomson, seconded by Mr. M. Van Raalte, stated that there were now twenty-three girls and twenty-two boys in attendance at the school, and the training college for teachers had been attended by nine students. Seven of the latter had obtained certificates. The committee regretted that they did not receive sufficient applications from students for training, and consequently they were unable to supply all the demands made on them for teachers. During the course of the afternoon a short illustration of tuition by the oral system was given, and afterwards Miss Amy Paget distributed the prizes to the pupils.

### THE PASSMORE EDWARDS CONVALESCENT HOME FOR THE METROPOLITAN HOSPITAL.

The Princess Louise opened the Convalescent Home recently built at the expense of Mr. Passmore Edwards, on land presented by Mr. F. S. W. Cornwallis, on Tuesday, July 20th. The arrangements for the reception of the Princess and the guests were very good. A special train conveyed the visitors to Staplehurst, where carriages awaited them, and took them to the home. The Princess on arrival inspected the home, after which several presentations were made to her. Mr. Passmore Edwards, jun., in the absence of his father, replying to the vote of thanks proposed by the chairman of the hospital board, Mr. C. T. Thomas, announced that Mr. Passmore Edwards would build a cottage hospital for the neighbourhood if a suitable site were given. Mr. Thomas said in the event of such a hospital being built, the Metropolitan Hospital would undertake to work it. Mr. Cornwallis replied to the vote of thanks to himself, bidding his hearers bear in mind that three acres in Kent was not quite so valuable as they would be in Belgravia. Her Royal Highness left the home soon after the ceremony, returning to town viâ Cranbrook, which was gaily decorated. The site of the new home is splendid. It stands high on a hill, from which wide and beautiful views are obtained over some of the fairest scenery in the Weald of Kent. The building is compact of one storey, and contains two portions identically the same, that to the right for men, that to the left for women convalescents. The entrance hall and matron's sitting-room occupy the centre, and a long corridor running parallel with the front of the building connects the two. Each side has its own staircase, and the similar corridor above is divided still more securely by a door which will be kept locked. The common dining hall juts out to the back, and has three doors—one for males, one for female convalescents, and one for domestics, communicating with kitchen, &c. Upstairs the same plan is followed. The ward above the dining-hall is arranged for six children; that above the women's day-room for six women's sleeping accommodation; that above the men's day-room for six men's sleeping accommodation. The matron's bed-room is next the men's ward, which she can view by means of a sliding shutter. Next her room is the nurses' bath-room, and next that, again, is the bed-room of the nurse in charge of the women convalescents, and communicating with the ward also by a shutter. The baths, lavatories, and offices are at either end of the long middle corridor, but shut off by glass doors. There are three sets of lavatories, &c., one for male, one for female, and one for children convalescents' use. They are practically in separate blocks, only joining the main building sufficiently to give covered access. The servants' accommodation is over the kitchen, and is reached by another flight of stairs. The bedrooms are arranged for sick nurses of the Metropolitan Hospital—one at either end of the long corridor on the ground floor and close by the men's and women's day-rooms respectively. The walls throughout are distempered sand-coloured above, with a dado of reddish terra cotta; the floors are carpetted with cork of a dark sandy hue, and protected where necessary by Oriental rugs. The furniture is sufficient, good, and that in the matron's and nurses' bed-rooms handsome. The cost was £500. The home cost nearly £3,000. Additional ventilation to that provided by windows and chimney was afforded in the sitting and day rooms by openings protected by mica valves passing into flues running up with the chimneys. In the bedrooms these flues were replaced by large ventilators in the ceilings opening into the roof, which was kept cool by free cross ventilation. The only room in which no provision was made of this kind was the mending room, and this had not a fireplace either.

The kitchen is a decidedly small one in which to prepare meals for 18 or 20 patients with a nursing staff of three, in addition to the necessary domestics. There is no other sitting room for the servants, and the heat when the large stone oil lamps are alight would be exhausting. Neither is the servants' bedroom large enough to give any relief in this particular; it is barely large enough for the two beds and furniture. The upper part of the window swinging open would give air enough if they were kept open, and there is a fireplace in which no fire could be lighted until some of the furniture is taken out. The registers of the stoves are tightly fitting, and the charwomen who had had things their own way so far had carefully shut every one, because of the dust that came down. Will the maids be wise enough to keep theirs open? It is a pity that in so compact and picturesque a new home these points should not have received due consideration. With regard to the servants' quarters, the architect needed more pecuniary freedom, but it is difficult to imagine a generous donor hesitating on the score of expense if the matter had been brought strongly to his notice.

### OPENING OF THE NEW BUILDINGS OF THE GROSVENOR HOSPITAL FOR WOMEN AND CHILDREN.

The Princess Louise, Marchioness of Lorne, opened these buildings on July 21st. This hospital occupies the site of the three old houses that have been hitherto used for hospital purposes. The patients were removed to 53, Vincent Square, during the rebuilding. The new hospital consists of a basement, ground floor, and two storeys. On the second storey are three general and two private wards, operating theatre, the ward kitchen, a bath-room, lavatories, &c. Shoots are provided for dust and soiled linen, and a lift for the provisions. The first floor is similarly arranged, and the ground floor has out-patients' department, doctors' consulting-rooms, dispensaries, &c. The out-patient department was arranged for the opening ceremony and tastefully decorated with flowers. The Princess was presented with a lovely bouquet by Miss Irene Ram, and, after the dedication service, with a golden key in a silver casket by the hon. architect (Mr. Roumieu). The building, which cost £9,000, is opened free of debt; but as it is not endowed subscriptions are invited for its maintenance. The Right Hon. Viscount Cross, G.C.B., G.C.S.I., took the chair; and amongst the guests present were: The Baroness Burdett-Coutts, Dean Bradley, Sir C. Freemantle, F. A. Bosanquet, Esq., Q.C.

